# Protein co-expression network analysis (ProCoNA)

**DOI:** 10.1186/2043-9113-3-11

**Published:** 2013-06-01

**Authors:** David L Gibbs, Arie Baratt, Ralph S Baric, Yoshihiro Kawaoka, Richard D Smith, Eric S Orwoll, Michael G Katze, Shannon K McWeeney

**Affiliations:** 1 Division of Bioinformatics and Computational Biology, Oregon Health & Science University, 3181 S.W. Sam Jackson Park Rd, Portland, OR 97239, USA; 2 Oregon Clinical & Translational Research Institute, Oregon Health & Science University, 3181 S.W. Sam Jackson Park Rd, Portland, OR 97239, USA; 3 Department of Microbiology and Immunology, University of North Carolina at Chapel Hill, 220 E Cameron Ave, Chapel Hill, NC 27514, USA; 4 Department of Pathobiological Sciences, University of Wisconsin-Madison, 2015 Linden Dr, Madison, WI 53706, USA; 5 Biological Sciences Division, Pacific Northwest National Laboratory, Richland, WA, USA; 6 Department of Microbiology, School of Medicine, Box 357735, University of Washington, Seattle, WA 98195, USA; 7 OHSU Knight Cancer Institute, Oregon Health & Science University, 3181 S.W. Sam Jackson Park Rd, Portland, OR 97239, USA

**Keywords:** Biomarkers, Biological networks, Networks, Systems biology, Virology, Sarcopenia, LC-MS, Proteomics

## Abstract

**Background:**

Biological networks are important for elucidating disease etiology due to their ability to model complex high dimensional data and biological systems. Proteomics provides a critical data source for such models, but currently lacks robust de novo methods for network construction, which could bring important insights in systems biology.

**Results:**

We have evaluated the construction of network models using methods derived from weighted gene co-expression network analysis (WGCNA). We show that approximately scale-free peptide networks, composed of statistically significant modules, are feasible and biologically meaningful using two mouse lung experiments and one human plasma experiment. Within each network, peptides derived from the same protein are shown to have a statistically higher topological overlap and concordance in abundance, which is potentially important for inferring protein abundance. The module representatives, called eigenpeptides, correlate significantly with biological phenotypes. Furthermore, within modules, we find significant enrichment for biological function and known interactions (gene ontology and protein-protein interactions).

**Conclusions:**

Biological networks are important tools in the analysis of complex systems. In this paper we evaluate the application of weighted co-expression network analysis to quantitative proteomics data. Protein co-expression networks allow novel approaches for biological interpretation, quality control, inference of protein abundance, a framework for potentially resolving degenerate peptide-protein mappings, and a biomarker signature discovery.

## Background

Systems biology embraces the complexity found in biological networks by taking a holistic view of the cell [1,2]. As systems biology moves forward, models making use of quantitative proteomic data will become increasingly necessary since this information is not accessible using other analytical methods [3,4].

Large-scale quantitative proteomics, however, is still developing and can be challenging and complex in practice [5,6]. In order to boost throughput and ease computation, tag-based approaches are used [7]. Briefly, proteins are digested enzymatically, producing a multitude of peptide fragments. Using liquid chromatography coupled to mass spectroscopy (referred to as LC-MS), the digested mixture is quantified, resulting in a set of features containing both mass and net elution time measurements. Peptides are identified by mapping features to entries in an accurate mass and time tag (AMT) database. Tag databases are constructed using pooled samples processed on a tandem MS/MS platform [8].

Currently, a majority of protein networks are constructed using protein-protein interaction (PPI) databases. However, manually curated PPI databases are regularly revised as our understanding of biology grows. PPI databases are typically heterogeneous, containing different experiment types and model organisms, leading to sparse annotation and a lack of experimental concordance [9]. In addition, interaction temporality and contextual information is lacking. Coverage, selection bias, and detection bias all remain problems [9,10].

**Figure 1:**
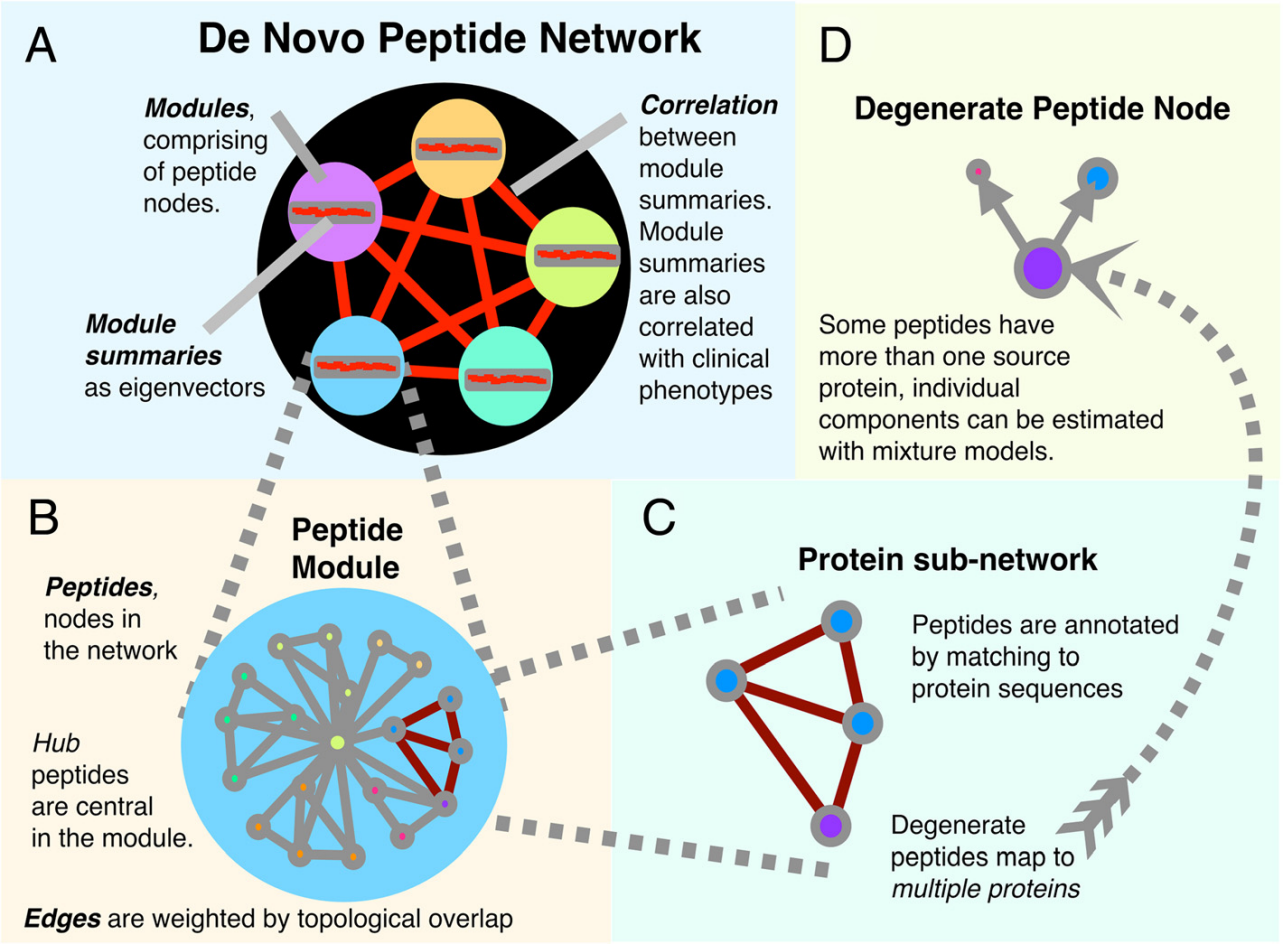
**Detail of the peptide network.** (**A**) The peptide network is decomposed into a set of subnetworks or modules. Module members, the peptide nodes, are more connected to peptides within the module, than across modules. (**B**) Singular value decomposition on the peptide abundance module matrix produces module summaries called eigenpeptides. Module prioritization is accomplished by correlating the eigenpeptide with sample phenotypes. By correlating peptides to the module summary, relative peptide importance is determined. (**C**) Taking peptides that map to a given protein defines a protein subnetwork. Peptides tend to be strongly connected within a protein subnetwork. (**D**) Difficulties arise when peptide nodes map to multiple proteins, rendering them degenerate.

De novo approaches based on observed data offer an alternative under which prior knowledge of protein interaction is eliminated and replaced by direct measurements of abundance. In this paper, we evaluate a novel approach to proteomic network analysis that is applicable to peptide and protein level data (see Figure 1). By using methods derived from weighted gene co-expression network analysis (WGCNA) [11,12], we show that unbiased de novo protein co-expression networks can be constructed and used for determining potential biomarkers, functional module prediction, and the discovery of important elements of human disease. We evaluate these methods utilizing data from two mouse infectious disease studies for SARS and Influenza, as well as a human population proteomics study for sarcopenia.

## Methods

### Mouse and human proteomic data sources

Three quantitative LC-MS data sets including both human and mouse disease studies were used. The Thermo Electron Exactive platform was used to generate data. The Pacific Northwest National Labs (PNNL, http://omics.pnl.gov) developed the Accurate Mass and Time (AMT) tag databases. VIPER (v3.48) is used to align individual samples and identify peptides using an AMT database [13]. Identifications have confidence metrics: the probability for a correct match, the STAC score, and the probability for a unique database match, the uniqueness probability (UP) [14]. Peptides with STAC scores > 0 and UP > 0 were used. Peptide abundances were normalized by total ion count per sample and log10 scaled. Missing data are encountered when peptides are identified in a subset of samples. “Missingness filtration” involves removing any peptide with greater than X% missing data across samples. In this case, peptides with greater than 10% missing data were removed.

The infectious disease data came from the NIAID Systems Virology project (publically available data is found at http://www.systemsvirology.org). We utilized both longitudinal SARS-CoV and influenza mouse studies. These data are generated using C57BL/6J mice exposed to either a mouse adapted SARS-CoV (MA-15) or avian influenza virus (A/Vietnam/1203/2004 (H5N1, VN1203)) [15,16]. Measurements took place on post infection days 1, 2, 4 and 7.

SARS control samples include three technical replicates per day. Infected samples are five technical replicates with viral dosages of 10^2^, 10^3^, 10^4^, and 10^5^ PFU per day. Abundance measurements for 16,890 peptides mapping to 3,277 proteins were recorded. After missingness filtration, 2,008 peptides mapping to 707 proteins remained. 352 proteins were associated with a single peptide, while 355 proteins had two or more peptides associated.

Influenza control samples include three technical replicates per day. Infected samples include five technical replicates with dosages of 10^2^, 10^3^, and 10^4^ PFU per day. Abundances for 10,285 peptides mapping to 2,661 proteins were recorded. After missingness filtration, 989 peptides associated with 493 proteins remained. 274 proteins were associated with a single peptide, while 219 proteins had at least 2 peptides associated.

The human proteomics data (currently unpublished) comes from a sub-cohort of participants selected from a large (N=6000) longitudinal study of musculoskeletal health in older (≥ 65 years) men (from the Osteoporotic Fractures in Men (MrOS) Study) [17,18]. The protocol was approved by the local institutional review boards. All participants provided written informed consent. The sub-cohort is focused on the sarcopenia phenotype, which is related to loss of lean mass and muscle performance [19]. A subset of 68 samples from two phenotyped groups (sarcopenic (N=38) and non-sarcopenic (N=30)) based on lean mass and leg power are used. Abundances for 10,679 peptides mapping to 1,868 proteins were recorded. After missingness filtration, 2,845 peptides mapping to 685 proteins remained. 505 proteins were associated with a single peptide, and 180 were associated with at least two peptides.

### Protein co-expression network construction

Protein co-expression networks contain nodes representing peptides connected with edges weighted by similarity in abundance profile. Edge weights are calculated using peptide intensity measurements. Although not always representative of absolute abundance, intensity is frequently used to track relative peptide abundance and to infer protein abundance [20]. In this work, we did not attempt to rectify situations where proteins were represented by a single peptide or where degenerate peptides mapped to multiple proteins. See Additional file 1 for a description of the software used in this work.

Construction of the network follows the WGCNA method [21-24]. Pearson’s correlations are computed pairwise between all peptides, retaining the sign as in Mason et al., resulting in a signed similarity matrix [25]. According to the scale-free criterion, a power (beta) is selected that transforms the distribution of node degrees in the similarity matrix to log-linear, producing the appropriate adjacency matrix. Topological overlap is a similarity metric that incorporates information from neighboring nodes, making it robust to noisy correlations. The TOM is computed as TOM_ij_ = (l_ij_ + a_ij_) / [min (k_i_, k_j_) + 1 - a_ij_] where l_ij_ is defined as the dot product on row i and column j in adjacency matrix [a] and k_i_ (the connectivity) is the summation of row i in adjacency matrix [a]. Modules, or subnetworks, are composed of strongly connected peptides. Modules are discovered by hierarchical clustering of the distance matrix, 1-TOM, using the “average” agglomeration method, followed by branch cutting with the dynamic hybrid treecut algorithm [21]. The following parameters were used after visualization and exploratory analysis: deepSplit = 2, minModuleSize = 30, mergeThreshold = 0.1.

### Calculating module significance using permutation testing

Similar to Iancu et al. [26,27], module significance was examined using permutation testing. Empirical p-values are computed by comparing the mean topological overlap of peptides within a module to a similarly sized random peptide sample. These samples are taken from the total set of peptides used in the network. For a given module with size n, mean edge weights are computed. For a number of trials, t, a sample of peptides is drawn with size equal to n, and the mean edge weight computed. If this value is equal to, or higher than the observed module mean, a count is incremented. The p-value is equal to (counts/t). In this work 10,000 random samples were drawn.

### Summarizing modules with eigenpeptides

After assigning peptides to modules, an aggregate module signature is computed. The first right-singular vector, or eigenpeptide, is computed from a singular value decomposition of the standardized abundance module matrix. The eigenpeptide has length equal to the number of samples. This vector acts as an overall summary of the module. Modules can be prioritized according to correlations between the eigenpeptide and biological phenotypes. Additionally, the relative centrality, or “importance” of any given peptide within a module is found by computing a Pearson’s correlation to the eigenpeptide (called the K_me_) [28,29]. Peptides with a strong correlation to the eigenpeptide are said to be more central, and important within the module, allowing prioritization on peptides.

### Describing concordance of peptides within modules

Concordance among a set of peptides relates to the shared sign of the slope when regressed against a given variable such as time or infection status. Our approach to this problem involves constructing protein sub-networks, initially as “all-to-all” networks. After applying a topological overlap threshold, edges start to fall away. This results in a disjoint set of connected components.

**Figure 2:**
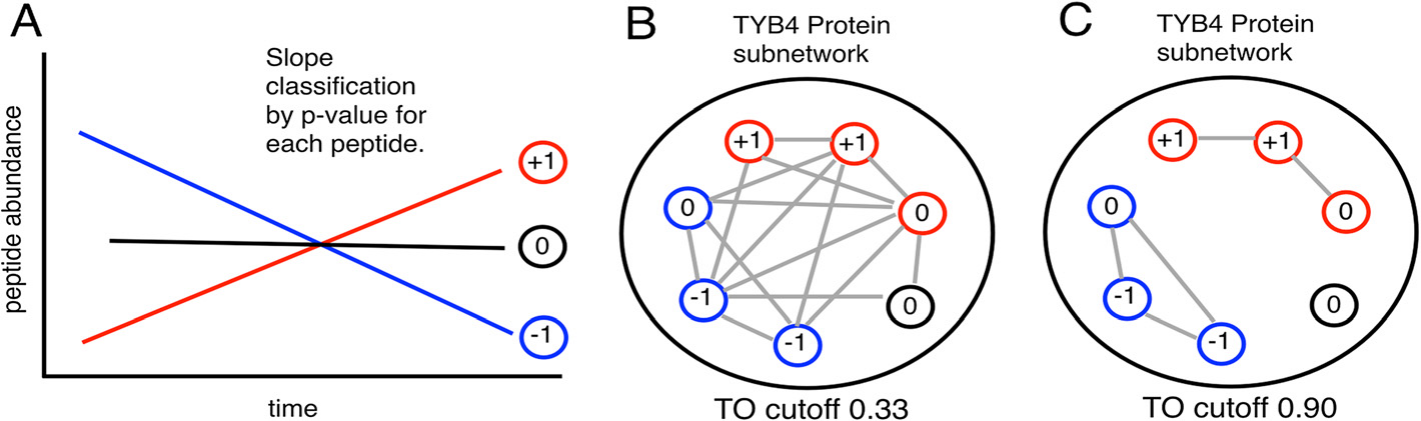
**Utility of de novo network inference in resolving peptide level discordance.** Protein sub-networks constructed using peptide topological overlap show correlated clusters. Taken together, some proteins show conflicts between constituent peptides, where given a variable such as time, some peptides are increasing in abundance and other decreasing (**A**). To examine this, only proteins with uniquely mapping peptides were used. A protein sub-network is constructed by taking associated peptides, and keeping only edges with topological overlaps in a specified upper quantile (e.g. the upper 20% of all topological overlaps for the protein). In all three data sources, as the edge threshold is raised, the number of connected components with discordant peptides dramatically decreases (**B**, **C**). This suggests that inference of protein abundance can be guided by network topology.

Two methods were used to examine whether concordant peptides are connected in the network. First, a linear model is constructed for each peptide using a reference variable such as time or a phenotypic trait. Peptides are classified as increasing (+1), decreasing (−1), or no-slope (0) depending on the adjusted p-value. If a connected sub-graph contains both increasing and decreasing peptides, it is considered a discordant component (see Figure 2).

Alternatively, the expression fold change (infected vs. non-infected) of peptides can be compared within a protein to determine discordance. A fold change cut-off of 1.5 was used to define peptides as up or down regulated. If both up and down labeled peptides mapped to a protein, then the protein was counted as discordant.

### Testing for strong peptide connectivity by protein

Similar to testing for module significance, the connectivity among peptides mapping to a given protein can be tested by permutation. For each protein with greater than two peptides, the pairwise topological overlaps are averaged. Then for a set number of trials (10,000), the same number of peptides is randomly sampled from all peptides in the network, and the mean pair wise topological overlap recorded. The empirical p-value is taken as the number of times the random sample has values equal or greater than the observed case divided by the number of trials. This test can also be applied using correlations between the peptides.

### Protein-protein interaction enrichment within modules

Co-expression modules are thought to reflect, to some degree, true protein interactions. To examine this, we compare the contents of modules with known PPIs. As previously done, permutation testing was used to determine whether a significant amount of PPI edges exist within a module. Within each module, peptides with weak connections to the module eigenpeptide were removed (K_me_ < 0.333). Centrality filtration is performed to focus the analysis on peptides associated with overall module function. The remaining peptides are mapped to proteins. Proteins with any number of mapping peptides are included. The number of observed PPIs within a module is recorded and compared to the number of PPIs in a random module for a set number of trials. P-values are computed using permutation testing as before. The PPI databases HPRD [30] and MPPI [31] were used for human and mouse data respectively.

### Pathway enrichment within modules

After PPI enrichment tests, significant sets of proteins were collected by module. Querying KEGG [32,33], using the R package KEGGSOAP [34], with these proteins provided a list of potential pathways to investigate by module. For each pathway returned, a hypergeometric test was performed using significant PPIs from the module and other proteins taking part in the pathway. The universe is defined as the subset of proteins in the mass tag database with known roles in KEGG pathways. P-values are adjusted using the Benjamini and Yekutieli method [35].

### Gene ontology functional enrichment within modules

Functional enrichment on modules was computed using the R package GOstats [36]. Peptides are mapped to proteins and counted once in any module. The universe is defined as all proteins found in the AMT mass tag database (similar to microarray studies). Annotation databases “org.Mm.eg.db” and “org.Hs.eg.db” (Bioconductor 2.8) are used for mouse and human annotations. Conditional hypergeometric testing is used to account for correlated GO terms.

## Results and discussion

### Peptide networks were approximately scale-free

**Figure 3:**
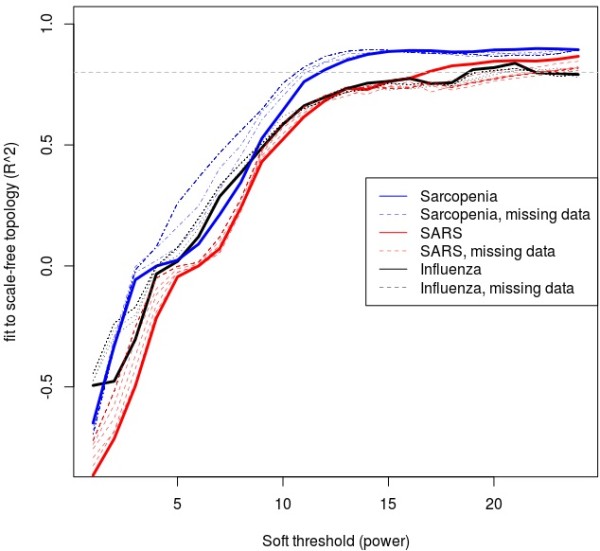
**Protein co-expression networks are shown to be approximately scale-free.** As the soft thresholding power, β, grows, the resulting adjacency matrix increasingly fits the scale-free model. This trend is robust to missing data, shown here with networks constructed using signed similarity matrices. Subsets of peptide data were taken to eliminate missing data (dark lines). Then, incrementally, additional peptides were included containing between one to ten missing data points, shown here with lighter, broken lines. For network analysis, it is strongly in our interest to incorporate peptides with missing data, since it increases the proteome coverage without weakening the model.

Scale-free network topologies have node degree distributions following the power law [37,38]. There is a continuous range of node degrees, with the fewest nodes having the greatest number of connections [37,38]. We found that peptide networks share this topology (Figure 3) and have biologically informative graph properties similar to those found in gene co-expression networks (See descriptive network statistics in Table 1).

**Table 1:** Co-expression network construction methods are applicable to proteomics

**Data**	**Peptides**	**Proteins**	**Power**	**R^2**	**Slope**	**Mean K**	**Modules**
**Sarcopenia**	2845	685	15	0.81	−1.55	25.22	19
**SARS**	2008	707	16	0.76	−1.67	10.8	14
**Influenza**	989	493	15	0.82	−1.31	7.00	6

Power is the parameter used to scale the adjacency matrix. *R*^*2*^*and Slope* describe the scale-free topology fit. Definition of mean K: network connectivity using the adjacency matrix.

With regard to distinct and significant modules, the SARS network contained 14 modules ranging from 65 to 369 peptides, with a mean size of 133.9 peptides. The Influenza network contained 6 modules, with sizes ranging from 56 to 327 peptides and a mean size of 141.3 peptides. The sarcopenia network contained 19 modules ranging in size from 36 to 477 peptides, with a mean size of 142.25 peptides. An initial examination indicates that low to moderate levels of missing data did not negatively impact the model fit (Figure 3). Importantly, we note that all of the identified de-novo modules have significant connectivity with the exception of the sarcopenia network, which contained one module without significant connectivity (p-value 0.33).

### Significant modules were correlated with phenotype

**Figure 4:**
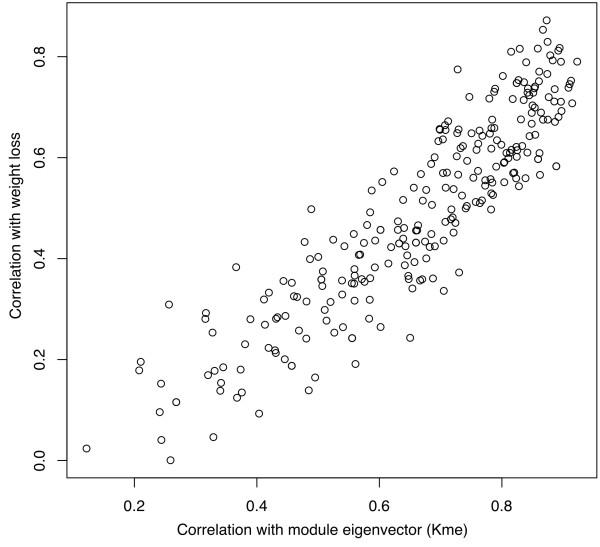
**Correlation with biological phenotypes can aid in prioritization of modules and proteins.** In modules where the eigenpeptide is strongly correlated with a biological phenotype, an upward trend is observed between the Kme of a peptide and the correlation with the given phenotype. An illustration from the Influenza data is shown. This demonstrates structural order within the module. After sorting along these dimensions, top peptides suggest further experiments.

Using module summaries (i.e., eigenpeptide), correlation with biological phenotypes can guide the discovery of biomarkers and aid in prioritization of modules for validation and perturbation experiments (see Figure 4).

**Figure 5:**
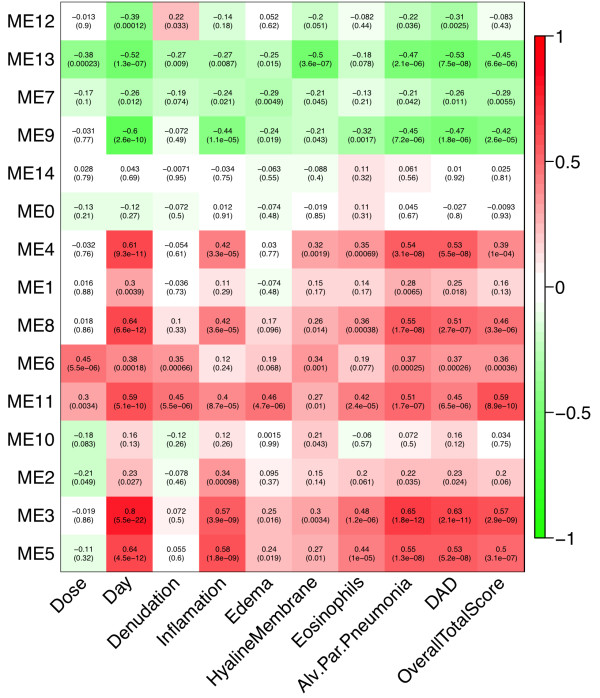
**The de novo modules (represented by module eigenpeptide, ME), are highly correlated to pathologically associated phenotypes.** An illustration from the SARS dataset is shown. Clear patterns emerge showing positive and negative correlation clusters. As expected, related phenotypes such as airspace inflammation, interstitial septum inflammation, and diffuse alveolar damage tend to be correlated in the same direction showing an overarching biological process at work. *Label Key: Alv.Par.Pneumonia: alveolar parenchyma pneumonia, DAD: diffuse alveolar damage, OverallTotalScore: cumulative score calculated by a pathologist.*

In the SARS network, strong correlations with disease-related pathological features were observed, including diffuse alveolar damage, tissue inflammation, and alveoli parenchyma pneumonia (Figure 5). The strongest correlations were found with time (module 3, Pearson correlation 0.8, p-value 1e-22) potentially relating to progression of infection.

The influenza network showed strong correlations with average weight loss, an important indicator of infection severity. Two modules showed positive correlation (p-values 2e-10 and 2e-6), and two modules showed negative correlation (p-values 8e-10 and 2e-15).

The sarcopenia network showed the weakest correlations with sample phenotypes. Several modules correlate with technical variables, indicating that the normalization method did not completely remove systematic effects. This finding guided the re-evaluation of data processing and motivated new methods in normalization, which is in preparation by Baraff et al.

### Peptide modules had significant protein-level connectivity

Given a complex biological mixture, a significant problem in quantitative proteomics remains in confidently identifying the protein component. This problem is made worse by the existence of degenerate peptides, which can lead to multiple solutions for peptide to protein mapping. We find that the connectivity of a protein’s constituent peptides is far from random. This is potentially useful for resolving cases of degenerate peptide mapping, increasing confidence in protein identification.

Topological overlap can be utilized with a threshold to identify high confidence edges between peptides. Upon examination, we found that with a topological overlap threshold of 80% (keeping only edges in the top 20% of all weights), the majority of proteins remained connected (Sarcopenia 84%, SARS 72%, influenza 63%).

To test for significant protein connectivity, we compared the mean topological overlap between constituent peptides and similar numbers of randomly selected peptides. In this evaluation of mean protein connectivities and random connectivities, we found significant connections between constituent peptides compared to those seen at random (Table 2). This suggests that the network structure should be helpful in resolving degenerate mappings by comparing network graphs and connectivities for alternative peptide-to-protein attributions.

**Table 2:** Network topology may be useful for resolving degenerate peptide mappings

**Data**	**Peptides**	**Proteins**	**Mean TO**	**RandomTO**	**p-value**
**Sarcopenia**	2845	685	0.089	0.004	2.09e-14
**SARS**	2008	707	0.025	0.004	2.2e-16
**Influenza**	989	493	0.028	0.005	8.99e-16

Results from a two-sample t-test between topological overlap (TO) of peptides derived from the same protein versus peptides selected at random. This result statistically shows that the connectivity for a protein’s constituent peptides is far from random. Network topology may be useful for resolving cases of degenerate peptide mapping, increasing confidence in protein identification.

### Strongly connected peptides were concordant

**Figure 6:**
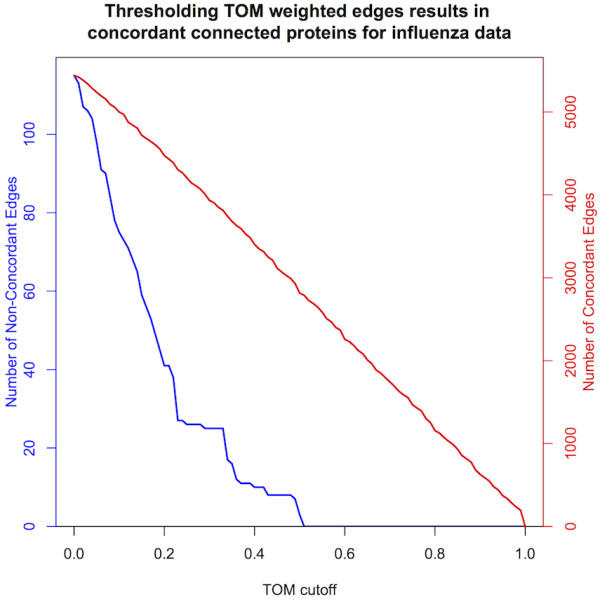
**Strongly connected peptides are concordant when regressed against time.** An illustration from the influenza dataset is shown. A protein sub-network consists of peptides mapping to a given protein, forming a all-to-all network with weighted edges. Applying an edge threshold decreases the number of “strong” peptide connections. An edge is counted as concordant if the two connected peptides have significant slopes in the same direction after linear regression against infection day. The discordant edges are clearly differentiated by edge thresholding.

Discordance observed among related peptides (i.e., those from the same protein) might reflect the activities of proteins with differing post-translational modifications or different isoforms. First we assessed the relationship between connectivity and peptide discordance with respect to abundance over time (Figure 6). In the influenza data set, as the edge weight threshold increases, the number of discordant edges quickly drops to zero far before the linear trend of concordant edges.

Examining discordance in the fold change of peptide expression, and using proteins with unique peptide mappings, 48 of 218 proteins in the SARS data were discordant. After applying a topological overlap threshold of 0.8 (as above), the number of discordant components dropped to 24, and with a threshold of 0.9 dropped to 12. In the sarcopenia network, peptides that were modeled against leg strength provided the most discordant proteins, resulting in 11 of 139 discordant proteins. After applying the topological overlap thresholds of 0.8 and 0.9, these dropped to 4 and 3 respectively. In influenza, there were 15 (out of 115 proteins) classified as discordant; this reduced to 7 and 3 respectively after applying the 0.8 and 0.9 thresholds. It appears that edge strength is informative with respect to concordance among constituent peptides for any given protein, showing potential utility for both protein inference and quantification.

### Modules had significant enrichment for known PPI interactions

De novo co-expression modules are thought to be useful for detecting new interactions and/or functional pathway members. We first evaluate, however, whether the de novo modules are enriched for known interactions to aid in assessing the utility of this approach. Using the HURD and MPPI protein-protein interaction databases, significant interactions were identified in all three experiments. After adjusting for multiple testing, the influenza network had 5/6 modules with significant PPI enrichment, the SARS network had 12/14 modules significantly enriched and the MrOS network had 10/19. Some modules overlapped in terms of mapped proteins which is typically the result of highly similar or related protein sequences, such as sets of histones.

We then examined whether modules with significant PPIs were also enriched for known pathways, similar to what has been seen in de-novo gene expression studies. For the influenza modules with significant PPI enrichment, we examined known pathways in the KEGG database. When defining the universe (or background for comparison) as all proteins contained in the mass tag database (5,521 proteins), a range of significant pathways were found, including “regulation of actin cytoskeleton” (mmu:04810), the “tight junctions” pathway (mmu:04530), and the “antigen presentation and processing” pathway (mmu:04612). However, if the universe is restricted to only those proteins with KEGG annotations (2,539 proteins), the antigen presentation pathway alone remained significant in two modules. These pathways are important in the pathological progression of influenza, highlighting the relationship between network structure and biology. Identification of enriched pathways in these unbiased modules could potentially aid discovery of novel interactions or pathway members.

### Modules had significant gene ontology functional enrichment

Given the significant numbers of PPI interactions, modules may have overarching functional organization. To study this, Gene Ontology enrichment, by module, was evaluated using the GOstats package [36]. All three data sources showed GO term enrichment with highly significant Bonferroni adjusted p-values (Additional file 2: Table S1). In the SARS and influenza networks, enrichment for biological processes such as DNA packaging, cellular component assembly, and cellular complex assembly was observed. The sarcopenia network modules also showed significant functional enrichment, including immune response and blood processes. This further reiterates the non-random, biological composition of these modules and provides support for the use of this approach to network inference in proteomics. We note that this framework is generalizable to many data types and is not limited to proteomics.

## Conclusions

We have demonstrated the feasibility of constructing de novo peptide co-expression networks. We show that these networks have a biologically meaningful and approximately scale-free topology and contain statistically significant modules. We also noted that the network structure and connectivity of the modules are potentially useful for resolution of degenerate peptides and inference of protein abundance. Across three distinct experiments, we have illustrated how module summaries significantly correlate with clinically relevant phenotypes. In addition, we have shown how de novo modules show significant enrichment for known PPI and biological function. Peptides can be ranked according to their module centrality and relationship to phenotypic traits, allowing researchers to prioritize targets for further research. Finally, modules can provide a natural aggregate representation for composite biomarker discovery.

## Competing interests

The authors declare that they have no competing interests.

## Authors’ contributions

DLG designed and programmed the methods, interpreted the results, and wrote the manuscript. AB maintained the proteomics pipeline and performed the peptide identification. RB provided the SARS-CoV virus and phenotype data. YK provided the influenza virus and phenotype data. RDS provided the proteomics data and software. EO provided data and interpretation of the human cohort. MK provided gene expression data. SM designed methods and made significant contributions to the writing. All authors read and approved the final manuscript.

## Supplementary Material

Additional file 1 The ProCoNA Software Supplemental (added to Bioconductor), describes the R package developed as part of this work, along with the relevant functions and descriptions of their use. Click here for file

Additional file 2: Table S1 Protein Co-expression Network Analysis (ProCoNA) GO enrichment summary results and examples. These tables give in-depth examples of the results from gene ontology enrichment for each experiment. Click here for file
