# Supplementary material for: Protein co-expression network analysis (ProCoNA)
Source: J Clin Bioinforma. 2013 Jun 1;3:11. doi: 10.1186/2043-9113-3-11 (PMC3695838; doi:10.1186/2043-9113-3-11)
Supplement: Additional file 1 — The ProCoNA Software Supplemental (added to Bioconductor), describes the R package developed as part of this work, along with the relevant functions and descriptions of their use. [file 2043-9113-3-11-S1.docx]

**Protein Co-expression Network Analysis (ProCoNA)**

Supplemental Materials: ProCoNA Software Package Description

Availability: The R package will be submitted to Bioconductor with simulated data.

Contact: David Gibbs, gibbsd@ohsu.edu

ProCoNA (protein co-expression network analysis) is an R package aimed at constructing and analyzing peptide networks. These networks are constructed using data derived from mass spectroscopy experiments (primarily LC-MS). This package streamlines the process of building networks using modern object oriented methods to bundle relevant information for downstream analysis. The package is built around calls to WGCNA functions (weighted gene co-expression network analysis), which are fast and robust. ProCoNA adds a suite of statistical tests particularly suited to the unique challenges found in proteomics. These tests include permutation testing for module structure and within-protein topological overlap. Peptide co-expression networks bring novel approaches for biological interpretation, quality control, inference of protein abundance, potentially resolving degenerate peptide-protein mappings, and biomarker signature discovery.

Functions:

newProconaObj:

This function returns a peptide co-expression network object containing all pertinent information.

toPermTest:

Topological Overlap Permutation Test. Uses the procona network object and the peptide data. Modules are permuted and mean topological overlap is recorded, constructing the null. The number of random permutations with mean TO greater than observed provides the p-value.

peptideConnectivityTest:

This function compares the connectivity between peptides mapped to a given protein, against a randomly drawn, similarly sized, selection of peptides. The hypothesis is that peptides from a given protein should be more connected than random.

peptideCorrelationTest:

This function compares the correlation between peptides mapped to a given protein, against a randomly drawn, similarly sized, selection of peptides.

goStatTest:

Wrapper function to run the hyperGTest from package GOstats on peptide modules.

ppiPermTest:

Performs a permutation test for enrichment of PPI edges given a database.

compareNetworksWithFishersExactTest:

This makes the heatmap of agreement between networks, module-wise, by comparing each pair of modules with Fisher's exact test.

correlationWithPhenotypesHeatMap:

Heatmap showing the correlation of modules summaries with phenotypes.
